# Impact of Capsaicinoid Supplementation in Health and Performance of Broiler Chickens Subjected to Lipopolysaccharide Challenge

**DOI:** 10.3390/ani15152203

**Published:** 2025-07-26

**Authors:** Rayanne A. Nunes, Kelly M. M. Dias, Marcio S. Duarte, Claudson O. Brito, Ricardo V. Nunes, Tiago G. Petrolli, Samuel O. Borges, Larissa P. Castro, Beatriz G. Vale, Arele A. Calderano

**Affiliations:** 1 Department of Animal Science, Federal University of Viçosa, Viçosa 36570-900, MG, Brazil; rayane.andrade9@gmail.com (R.A.N.); marcio.duarte@ufv.br (M.S.D.); samuel.borges@ufv.br (S.O.B.); larissa.p.castro@ufv.br (L.P.C.); beatriz.vale@ufv.br (B.G.V.); 2 Department of Animal Biosciences, University of Guelph, Guelph, ON N1G 2W1, Canada; 3 Department of Animal Science, Federal University of Sergipe, São Cristóvão 49100-000, SE, Brazil; claudson@academico.ufs.br; 4 Department of Animal Science, State University of Western Paraná, Marechal Cândido Rondon 85960-128, PR, Brazil; nunesrv@hotmail.com; 5 Department of Animal Science, University of the West of Santa Catarina, Xanxerê 89820-000, SC, Brazil; tiago.petrolli@unoesc.edu.br

**Keywords:** feed additive, inflammation, antioxidant, poultry

## Abstract

This study investigated the effects of dietary capsaicinoid (CAP) supplementation on broiler chickens subjected to an inflammatory challenge induced by lipopolysaccharide (LPS). A total of 144 Cobb500™ male broilers (Rivelli Alimentos SA, Matheus Leme, Brazil), raised from 1 to 21 days, were randomly assigned to three treatments, with eight replicates of six birds. Treatments were a control diet (CON), a control diet with LPS administration (CON+LPS), and a control diet supplemented with 1 mg CAP/kg feed and LPS (CAP+LPS). LPS was administered intraperitoneally on days 14, 16, 18, and 20. Performance, intestinal morphometry, serum metabolites, and jejunal gene expression related to oxidative and inflammatory responses were evaluated. Slaughter was at 20 days. Data were subjected to ANOVA and means compared by Tukey’s test at 0.05 significance. CON broilers exhibited the highest feed intake and a better feed conversion ratio (*p* < 0.05) compared to CON+LPS. CAP+LPS broilers showed higher body weight gain than CON+LPS but lower than CON broilers (*p* < 0.001). CON+LPS broilers had the highest crypt depth (*p* = 0.002). Higher mRNA expression of superoxide dismutase and catalase (*p* > 0.05) was observed in CON broilers. In conclusion, supplementation with a 1 mg CAP/kg diet improves the growth performance and intestinal morphometry of LPS-challenged broiler chickens.

## Simple Summary

In modern poultry farming, chickens are often exposed to conditions that cause inflammation and stress, such as infections or environmental challenges. These stressors reduce growth and impair gut function, leading to economic losses and lower animal welfare. In this study, we evaluated whether capsaicinoids—natural compounds responsible for the spiciness of chili peppers—could help protect chickens during an immune challenge. To simulate an inflammatory condition, we injected lipopolysaccharide to trigger immune responses. A total of 1 mg capsaicinoids/kg feed was added to the chickens’ feed to test if it could reduce the damage caused by inflammation. Our findings showed that chickens receiving capsaicinoids had better weight gain and improved intestinal structure compared to those that were only exposed to the immune challenge. These results suggest that dietary capsaicinoids may offer partial protection to chickens under immune stress by helping maintain intestinal health and growth and could contribute to more resilient broiler chickens.

## 1. Introduction

In practical production systems, broilers are frequently subjected to inflammatory challenges, which harm their overall performance. Pro-inflammatory mediators are known to suppress appetite, leading to reduced nutrient intake. The equilibrium between nutrient allocation for immune response and potential reduction in the feed intake (FI) contributes to the diminished availability of nutrients meant for growth, potentially inducing tissue catabolism [1,2].

Capsaicinoids (CAP) are compounds naturally present in some species of peppers, and they are receiving increasing amounts of attention due to their bioactive properties and the potential effects they can have on the immune response of piglets and broilers [3,4,5,6]. Among capsaicinoids, capsaicin and dihydrocapsaicin are the primary molecular components, representing approximately 90% of the total CAP in peppers [7,8,9]. CAP are characterized by their antioxidant, anti-inflammatory, antimicrobial, and microbiota modulation activities [4,6,10]. Studies have also indicated that these molecules can decrease lipid peroxidation and increase nutrient digestibility, possibly because the production of digestive enzymes is stimulated [6,11,12,13]. Furthermore, CAP has been shown to play an important role in mitigating the stress response in poultry [14,15] and improving the FI, feed conversion rate (FCR), and carcass characteristics [12,13,16].

Investigating the effects of CAP is particularly important in situations where birds are exposed to stress and inflammatory challenges—factors that can lead to excessive activation of nuclear factor-κB (NF-κB), resulting in chronic inflammation, compromised health, and reduced productive performance [17,18]. In experiments with broilers, lipopolysaccharide (LPS), an integral component of the cell wall of Gram-negative bacteria, has been widely used to trigger the inflammatory response [16,19,20,21]. LPS-challenged broilers exhibit an acute inflammatory response and oxidative damage, characterized by reduced expression and activity of antioxidant enzymes, along with elevated production of pro-inflammatory cytokines [22,23].

Thus, in this study, we hypothesize that CAP has anti-inflammatory properties and that its use as a dietary supplement can mitigate the decrease in broiler performance caused by inflammatory challenge. Therefore, we aimed to evaluate the effect of dietary supplementation with CAP on performance, intestinal morphometry, and gene expression of broiler chickens challenged with lipopolysaccharide (LPS).

## 2. Materials and Methods

### 2.1. Ethical Issues

All procedures involving animals were approved by the Animal Ethics Committee of the Federal University of Viçosa, Brazil (protocol no. 39/2021; approval date: 9 July 2021). The trial followed the guidelines established by the Brazilian College of Animal Experimentation for the ethical use of live poultry in research.

### 2.2. Birds, Experimental Design, and Diets

For this study, 144 one-day-old male Cobb500™ broiler chicks were acquired from a commercial hatchery (Rivelli Alimentos SA, Matheus Leme, MG, Brazil). Birds were vaccinated against Gumboro and Marek (serotype 3, live Marek’s disease vector, Merial Inc., Athens, GA, USA). From 1 to 7 days of age, the birds were raised in protective circles according to lineage management recommendations.

On day 8 of age, birds were weighed (192.1 ± 66 g) and randomly assigned to an experimental design consisting of three treatments, eight replicates, and six birds per experimental unit. The birds were housed in wire cages (500 cm^2^/bird) equipped with trough-type feeders and nipple-type drinkers, totaling 24 experimental units. The treatments were a control diet (CON), the control diet and LPS administration (CON+LPS), and a control diet supplemented with 1 mg CAP/kg diet and LPS administration (CAP+LPS). CAP were provided using Capcin^®^ (ID4Feed, Annecy, France), containing 5 g of CAP per kg of product, and included in the diet at a concentration of 200 mg/kg. A corn–soybean meal basal diet was formulated according to the nutritional requirements described by Rostagno et al. [24] (Table 1). Diets were offered in mash form, and birds had free access to feed and water throughout the experimental period (from 8 to 21 days of age).

Birds in the CON+LPS and CAP+LPS treatment groups received intraperitoneal injections of *Escherichia coli* LPS (serotype O55:B5, Sigma Chemical Co., St. Louis, MO, USA), diluted in saline solution at a concentration of 1.0 mg/mL, at 14, 16, 18, and 20 days of age, administered in the lower abdominal quadrant. The initial dose was 1 mg/kg body weight; this was increased by 12% in subsequent applications [25]. Birds in the CON treatment received a similar amount of saline solution.

During the experimental period, the ambient temperature was maintained at 22 °C, and birds were subjected to a daily photoperiod of 18 h of continuous light.

### 2.3. Performance and Sample Collection

At the beginning and end of the experimental period (8 and 21 days of age, respectively), the birds and their feed were weighed. These data were used to calculate the body weight gain (BWG), FI, and FCR. In cases of mortality, the remaining feed was weighed to adjust the FI accordingly.

At 20 days, the bird with the weight closest to the average weight of the experimental unit was chosen for sample collection. Four hours after the LPS application, blood was drawn from the wing vein of the same bird and centrifuged at 3600× *g* for 10 min at 4 °C to obtain the serum, which was stored at −20 °C until analysis. Following blood sampling, the bird was euthanized via cervical dislocation. A jejunal segment of approximately 2 cm was excised, placed in a cryogenic tube, and immersed in liquid nitrogen. The samples were then transferred to a −80 °C freezer, where they remained until RNA extraction. Another segment of jejunum was also collected to determine the villus height (VH), crypt depth (CD), and their ratio (VH:CD). The lymphoid organs (bursa of Fabricius and spleen) were removed and weighed separately on a digital scale (0.0001 g) to determine their weight relative to the live weight of the animal.

### 2.4. Serum Parameter Measurements

Serum concentrations of glucose and triglycerides were determined using an automated biochemical analyzer (Cobas c 311; Roche Diagnostics GmbH, Basel, Switzerland), in accordance with the manufacturer’s protocol. For the determination of malondialdehyde (MDA) levels, 0.5 mL of serum was mixed with 2.5 mL of 20% trichloroacetic acid and 1.0 mL of 0.67% thiobarbituric acid. The mixture was then incubated in boiling water for 30 min. After cooling, 4.0 mL of n-butyl alcohol was added for chromogen extraction, and the absorbance of the organic layer was measured at 530 nm.

### 2.5. Determination of mRNA Content

Total RNA was extracted from 50 mg of jejunal tissue using TRIzol^®^ reagent (Invitrogen, Carlsbad, CA, USA) according to the manufacturer’s protocol. The RNA pellet was resuspended in 25 μL of UltraPure DNase/RNase-free water. Concentration and purity were determined using a NanoDrop™ Lite Spectrophotometer (Thermo Fisher Scientific, Beverly, MA, USA), and the RNA integrity was verified by electrophoresis on a 1.0% agarose gel. First-strand cDNA synthesis was performed using the High-Capacity cDNA Reverse Transcription Kit (Thermo Fisher Scientific, Beverly, MA, USA). Primer sequences are shown in Table 2. The reference gene β-actin was used for normalization due to its high expression stability. The expression levels of nuclear factor-κB (NF-κB), interleukin 1β (IL-1β), interleukin 10 (IL-10), glutathione peroxidase (GPx), catalase (CAT), and superoxide dismutase (SOD) were evaluated. Quantitative RT-PCR was performed in duplicate on an Applied Biosystems™ QuantStudio Real-Time PCR System (Thermo Fisher Scientific, Beverly, MA, USA) using the SYBR^®^ Green detection system and GoTaq^®^ qPCR Master Mix (Promega Corporation, Madison, WI, USA). The cycling conditions consisted of an initial denaturation at 95 °C for 2 min, followed by 40 cycles of 95 °C for 15 s and 60 °C for 1 min. Threshold cycle (Ct) values were normalized against β-actin (ΔCt), and relative expression was calculated using the 2^−ΔCt^ method, as described by [26].

### 2.6. Intestinal Morphometry

The 2 cm jejunum samples were washed in saline and kept in 10% formaldehyde phosphate buffer for 48 h. Cross-sections were then prepared, and the segments were dehydrated in a graded ethanol series, diaphanized with xylene, and embedded in liquid paraffin at 60 °C. Paraffin blocks were fixed in a rotating microtome (Spencer^®^ model 19459, Nova York, NY, USA), and the transverse sections were sliced to a thickness of 5 μm (the sections were made semi-serially, 1 in each of the 10 sections to avoid repeating the analyses in the same histological area). Six sections were placed on each glass slide and stained with hematoxylin–eosin. Five slides were prepared from the jejunal segment of each bird: 10 well-oriented villi were measured per slide (50 villi per bird). The sections were examined under an optical microscope (EVOS^®^ XL Core Imaging System, Thermo Fisher Scientific Inc., Bothell, WA, USA) at 10× magnification. Morphometric analysis was performed using ImageJ software (version 1.49, National Institutes of Health, Bethesda, MD, USA). The VH was measured from the top of the villi to the junction of the villus with the crypt, and the CD was measured from the base of the villus to the submucosa. The VH:CD ratio was calculated.

### 2.7. Statistical Analysis

Data were subjected to one-way ANOVA using the GLM procedure of SAS (Statistical Analysis System, 9.4), with subsequent comparison between means using the Tukey test. Differences were considered significant when *p* < 0.05. A replicate was the experimental unit for performance parameters, and the individual broiler from each replicate was the experimental unit for other data.

## 3. Results

### 3.1. Performance

Broilers subjected to the CAP+LPS treatment showed a higher BWG than those subjected to the CON+LPS treatment and lower than the CON treatment (*p* < 0.001; Table 3). Broilers in the CON treatment exhibited the highest FI (*p* = 0.011) and better FCR than those in the CON+LPS group (*p* = 0.022).

### 3.2. Relative Weight of Organs

The LPS challenge, independent of the supplementation of CAP, increased the relative spleen weight of the broilers (*p* < 0.001; Table 4). Treatments had no significant effect on the relative weight of the bursa of Fabricius (*p* > 0.05).

### 3.3. Serum Metabolites

The treatments had no significant effect on the serum levels of glucose, triglycerides, or cholesterol (*p* > 0.05; Table 5).

### 3.4. Intestinal Morphometry

Broilers that received the CON+LPS treatment showed higher CD than those in the CON and CON+LPS treatments (*p* = 0.002; Table 6). The VH:CD ratio was higher in the CON broilers (*p* = 0.022) than in the CON+LPS treatment. However, the treatment did not significantly affect VH (*p* > 0.05).

### 3.5. Relative mRNA Expression of Markers

**Figure 1:**
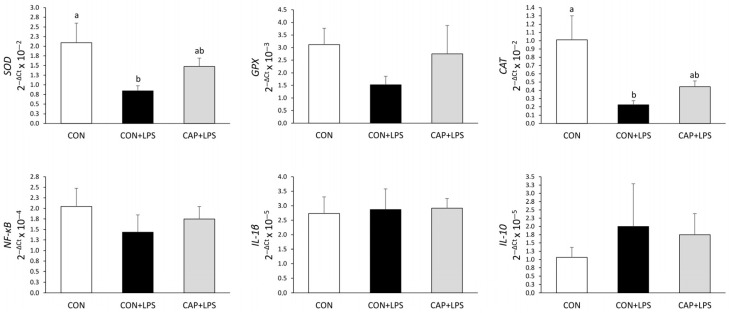
The mRNA expression of superoxide dismutase (SOD), glutathione peroxidase (GPx), catalase (CAT), nuclear factor-κB (NF-κB), interleukin 1 (IL-1β), and interleukin 10 (IL-10) in the jejunum of broilers at 20 days of age, 4 h after LPS administration. Comparisons were made between the control diet (CON), control diet and LPS administration (CON+LPS), and control diet supplemented with 1 mg capsaicinoids/kg diet and LPS administration (CAP+LPS). n = 8 per treatment. Bars (±standard error of the mean) with different letters differ by the Tukey test. *p*-values: SOD *p* = 0.046; GPx *p* = 0.335; CAT *p* = 0.012; NF-κB *p* = 0.546; IL-1β *p* = 0.971; IL-10 *p* = 0.726.

A higher mRNA expression of SOD (*p* = 0.046) and CAT (*p* = 0.011; Figure 1) was observed in the jejunum of the CON broilers than in broilers of the CON+LPS treatment. However, treatment did not affect the mRNA expression of IL-10, IL-1β, GPx, and NF-κB (*p* > 0.05).

## 4. Discussion

The hypothesis of this study was that CAP possess anti-inflammatory properties and that their dietary supplementation could mitigate the negative effects of an inflammatory challenge on broiler chickens. To test this, we used a well-established model of systemic inflammation induced by intraperitoneal LPS injection [19,20,21,27], which is known to impair performance and intestinal morphology through the redistribution of nutrients toward the immune system and by activating stress pathways in the gut [17,28]. LPS is an integral component of the cell wall of Gram-negative bacteria. It is a microbial-associated molecular pattern (MAMP) and potent stimulator of inflammation. The immune cells from the gut, likely phagocytes such as dendritic cells, identify this MAMP and quickly stimulate the production of pro-inflammatory cytokines, further amplifying the response by the recruitment of other leukocytes and soluble factors from the blood into the affected tissue. The inflammatory response results in stimulation of stress pathways, and particularly to LPS, which is known to have a toxic effect on lymphocytes (e.g., T cells). Furthermore, inflammatory stress induced by LPS injection causes a reduction in the FI in broilers, which might be due to the anorexia response through the control of the hypothalamic–pituitary–adrenal axis [29,30].

In our study, the LPS challenge successfully reduced the FI and BWG, and worsened the FCR, confirming the induction of inflammatory stress. LPS also affected intestinal morphometry, increasing CD and reducing VH:CD, consistent with previous findings that associate inflammation with impaired absorptive function and increased enterocyte turnover [31,32]. This may partly explain the reduction in performance observed.

Supplementation with CAP partially reversed these negative effects, where BWG was improved compared to the CON+LPS group, and CD was significantly reduced, suggesting a beneficial effect of CAP on intestinal morphometry under inflammatory conditions. VH and CD are important indicators of the structural integrity of the intestinal mucosa and intestinal digestion and absorption function. Crypts are the site of new enterocyte multiplication [31], and a larger CD is associated with worse intestinal quality and a high intestinal renewal rate [32]. This was the only finding that could explain the improvement in the BWG of the birds on diets supplemented with CAP. However, performance in the CAP+LPS group did not reach the same level as the unchallenged control group, indicating that the effect of CAP at this dosage is limited. Li et al. [12] have identified improvements in the FCR of broilers supplemented with 2 and 4 mg capsaicin/kg and attributed part of this result to the enhanced VH, villus width, and villous surface area in the jejunum of the broilers, suggesting that capsaicin improved the utilization of nutrients.

The assessment of lymphoid organ weights is a crucial indicator of the immune response of birds, providing valuable insights into their overall health (Dias; Tong; Wang) [21,33,34]. The spleen is a lymphoid organ, and its B cells produce antibodies in response to antigenic stimulation [35]. LPS can directly activate B cells through TLR4 receptors, leading to proliferation and differentiation into antibody-secreting plasma cells. In the present study, the LPS challenge induced spleen enlargement in broilers, confirming the activation of the immune system. This result is similar to the observations of Chen et al. [19] and Dias et al. [21], highlighting the importance of this organ and the increase in the metabolism of the chickens during the acute-phase inflammatory immune response, resulting in its mass growth. However, supplementation with CAP at the dose studied did not reduce this effect.

Regarding the serum metabolites of the broilers, the LPS injections were expected to induce hypolipidemia and hypoglycemia [28]. In addition, an increase in MDA was expected in association with the LPS challenge [36]. MDA is a key indicator of lipid peroxidation, with elevated concentrations reflecting increased oxidative stress in the organism [37]. However, in the present study, the LPS challenge did not influence the serum levels of glucose, triglycerides, or MDA. Furthermore, CAP supplementation did not affect the levels of these metabolites in the serum of the broilers. This result is similar to those reported by Kreuz et al. [4] with supplementation of 1 and 2 mg of CAP/kg feed. This contrasts with a recent study where Zanotto et al. [16] observed a reduction in thiobarbituric acid levels in the serum and breast meat of turkeys fed diets supplemented with 4 mg CAP/kg feed.

Oxidative stress can compromise cellular integrity by promoting lipid peroxidation, protein and DNA damage, and apoptotic processes [38]. However, the burden of reactive oxygen species (ROS) production can be counteracted by an antioxidant defense system, including the enzymes SOD and CAT. The LPS challenge normally increases the production of ROS in broilers and suppresses the activity of SOD and CAT in the serum, liver, and total antioxidant capacity in broilers [34,36]. Contrary to this, CAP supplementation did not restore the mRNA expression of SOD and CAT, both of which were reduced by the LPS challenge. These results contrast with those of Kreuz et al. [4], who reported that supplementation with 1 or 2 mg CAP/kg of diet enhanced SOD expression in the jejunum of broilers. However, the differences in antioxidant capacity in response to CAP supplementation may be attributed to the differing ages of the broilers in that study compared to the present one.

The inflammatory response of broilers can be measured from the increase in the production of NF-κB and by increased serum concentrations of cytokines, such as TNF-α, IL-1β, IL-6, and IL-10 [39]. However, in the present study, neither the LPS challenge nor CAP supplementation changed the mRNA expression of NF-κB, IL-1β, or IL-10 in the jejunum of the broilers. These results also contrast with Kreuz et al. [4], who reported a reduction in the expression of NF-κB mRNA in the jejunum of broiler chickens receiving 1 and 2 mg of CAP/kg diet. In addition, Liu et al. [40] observed lower levels of the pro-inflammatory cytokine IL-1β in the serum of broilers fed a natural capsaicin extract.

## 5. Conclusions

Dietary supplementation with 1 mg CAP/kg feed partially mitigates the negative effects of LPS-induced inflammation in broiler chickens. CAP improves the growth performance and jejunal morphometry of LPS-challenged broilers. However, this level of supplementation does not influence the expression of genes related to oxidative and inflammatory stress in the jejunum of these birds. Although the levels of capsaicin supplementation in this study did not influence the expression of genes related to oxidative and inflammatory stress in the jejunum of the broilers, future studies should assess capsaicin supplementation under heat stress conditions. Heat stress is a global issue in poultry farming, and as climate change intensifies, it is important to research ways to mitigate its negative effects in poultry production systems, using nutritional interventions. Overall, this study provides valuable information that will serve as a basis for future studies assessing the impact of capsaicin supplementation in poultry.

## Figures and Tables

**Table 1:** Ingredients and nutritional composition of basal diets (g/kg), as-fed basis.

	8 to 21 Days of Age
Corn ^3^	503.7
Soybean meal ^3^	411.2
Soybean oil	45.8
Dicalcium phosphate	16.8
Limestone	8.4
Salt	5.2
DL-Methionine ^3^	3.2
L-Lysine HCl ^3^	1.5
Vitamin premix ^1^	1.3
Trace mineral premix ^2^	1.2
Choline chloride ^3^	1.0
L-Threonine ^3^	0.6
L-Valine ^3^	0.1
Calculated composition	
Metabolizable energy, MJ/kg	12.76
Crude protein	230.0
Calcium	8.78
Available phosphorus	4.19
Sodium	2.18
Arginine	14.50
Digestible lysine	12.56
Digestible methionine + cysteine	9.29
Digestible threonine.	8.29
Digestible tryptophan	2.65
Digestible valine	9.67

^1^ Vitamin premix provided per kg of product: vitamin A, 12,528 UI; vitamin D3, 3132 UI; vitamin E, 46.9 UI; vitamin K3, 2.51 mg; vitamin B1, 3.37 mg; vitamin B12, 0.021 mg; vitamin B6, 4.69 mg; vitamin B5, 16.8 mg; vitamin B3, 51.0 mg; vitamin B9, 1.17 mg; biotin, 0.12 mg. ^2^ Trace mineral premix provided per kg of product: Mn, 70.03 mg; Zn, 65.05 mg; Fe, 50.01 mg; Cu, 9.97 mg; I, 1.012 mg; Se, 0.3 mg. ^3^ Corn (7.86% CP), soybean meal (45% CP), DL-Methionine (99.9%), L-Lysine HCl (78.0%), choline chloride (60%), L-Threonine (98.5%), and L-Valine (99.0%) were used as commercial sources.

**Table 2:** Primer sequences for quantitative reverse transcription-PCR.

Gene	Forward Sequence	Reverse Sequences
*NF-κB*	GTGTGAAGAAACGGGAACTG	GGCACGGTTGTCATAGATGG
*IL-1β*	GCTCTACATGTCGTGTGTGATGAG	TGTCGATGTCCCGCATGA
*IL-10*	CATGCTGCTGGGCCTGAA	CGTCTCCTTGATCTGCTTGATG
*GPx*	GACCAACCCGCAGTACATCA	GAGGTGCGGGCTTTCCTTTA
*SOD*	AGGGGGTCATCCACTTCC	CCCATTTGTGTTGTCTCCAA
*CAT*	ACTGCAAGGCGAAAGTGTTT	GGCTATGGATGAAGGATGGA
*β-actin*	TGCTGTGTTCCCATCTATCG	TTGGTGACAATACCGTGTTCA

**Table 3:** Feed intake (FI), body weight gain (BWG), and feed conversion ratio (FCR) of broiler chickens from 8 to 21 days of age.

	CON	CON+LPS	CAP+LPS	SEM	*p*-Value
FI (kg/bird)	1.031 ^a^	0.974 ^b^	1.005 ^ab^	0.008	0.011
BWG (kg/bird)	0.679 ^a^	0.611 ^c^	0.647 ^b^	0.006	<0.001
FCR	1.52 ^b^	1.59 ^a^	1.55 ^ab^	0.01	0.022

SEM: standard error of means (n = 8 for each treatment). Means on the same line, followed by different letters, differ from each other by the Tukey test (*p* < 0.05).

**Table 4:** The relative weight of lymphoid organs of broiler chickens at 20 days of age.

	CON	CON+LPS	CAP+LPS	SEM	*p*-Value
Bursa (%)	0.180	0.180	0.184	0.007	0.070
Spleen (%)	0.090 ^b^	0.165 ^a^	0.174 ^a^	0.009	<0.001

SEM: standard error of means (n = 8 for each treatment). Means on the same line, followed by different letters, differ from each other by the Tukey test (*p* < 0.05).

**Table 5:** Serum metabolites of broiler chickens at 20 days of age.

	CON	CON+LPS	CAP+LPS	SEM	*p*-Value
MDA (nmol/mL)	2.43	2.57	2.53	0.08	0.793
Glucose (mg/dL)	231.2	235.6	230.8	5.1	0.922
Triglycerides (mg/dL)	43.75	47.00	41.62	2.92	0.767

mL: milliliters; dL: deciliters. SEM: standard error of means (n = 8 for each treatment).

**Table 6:** Villus height (VH), crypt depth (CD), and VH:CD ratio in the jejunum of broiler chickens at 20 days of age.

	CON	CON+LPS	CAP+LPS	SEM	*p*-Value
VH (μm)	935.9	887.9	914.9	21,0	0.664
CD (μm)	195.2 ^b^	236.9 ^a^	201.2 ^b^	5.8	0.002
VH:CD (μm)	4.90 ^a^	3.77 ^b^	4.56 ^ab^	0.17	0.022

SEM: standard error of means (n = 8 for each treatment). Means on the same line, followed by different letters, differ from each other by the Tukey test (*p* < 0.05).

## Data Availability

The data are available in the manuscript; there is no external repository.
